# Electrochemical Immunosensing of ST2: A Checkpoint Target in Cancer Diseases

**DOI:** 10.3390/bios11060202

**Published:** 2021-06-21

**Authors:** Rebeca M. Torrente-Rodríguez, Cristina Muñoz-San Martín, Maria Gamella, María Pedrero, Neus Martínez-Bosch, Pilar Navarro, Pablo García de Frutos, José M. Pingarrón, Susana Campuzano

**Affiliations:** 1 Departamento de Química Analítica, Facultad de CC. Químicas, Universidad Complutense de Madrid, 28040 Madrid, Spain; rebecamt@ucm.es (R.M.T.-R.); cmunoz04@ucm.es (C.M.-S.M.); mariagam@quim.ucm.es (M.G.); pingarro@quim.ucm.es (J.M.P.); 2 Cancer Research Program, Hospital del Mar Medical Research Institute (IMIM), Unidad Asociada IIBB-CSIC, 08003 Barcelona, Spain; nmartinez@imim.es (N.M.-B.); PNavarro@imim.es (P.N.); 3 Departamento de Muerte y Proliferación Celular, Instituto de Investigaciones Biomédicas de Barcelona–Centro Superior de Investigaciones Científicas (IIBB-CSIC), 08036 Barcelona, Spain; pablo.garcia@iibb.csic.es; 4 Institut d’Investigacions Biomédiques August Pi i Sunyer (IDIBAPS), 08036 Barcelona, Spain

**Keywords:** electrochemical immune platform, human ST2, plasma, pancreatic cancer

## Abstract

A magnetic beads (MB)-involved amperometric immunosensor for the determination of ST2, a member of the IL1 receptor family, is reported in this work. The method utilizes a sandwich immunoassay and disposable screen-printed carbon electrodes (SPCEs). Magnetic immunoconjugates built on the surface of carboxylic acid-microsized magnetic particles (HOOC-MBs) were used to selectively capture ST2. A biotinylated secondary antibody further conjugated with a streptavidin peroxidase conjugate (Strep-HRP) was used to accomplish the sandwiching of the target protein. The immune platform exhibits great selectivity and a low limit of detection (39.6 pg mL^−1^) for ST2, allowing the determination of soluble ST2 (sST2) in plasma samples from healthy individuals and patients diagnosed with pancreatic ductal adenocarcinoma (PDAC) in only 45 min once the immunoconjugates have been prepared. The good correlation of the obtained results with those provided by an ELISA kit performed using the same immunoreagents demonstrates the potential of the developed strategy for early diagnosis and/or prognosis of the fatal PDAC disease.

## 1. Introduction

IL1RL1/IL33R encodes a member of the interleukin 1 receptor family known as ST2 (or IL-33R), which consists of a transmembrane receptor (ST2L) and truncates soluble (sST2) isoforms. ST2 has well-known relations with inflammatory diseases; elevated circulating levels of sST2 are found in the serum of patients suffering from several disorders such as systemic lupus erythematosus pulmonary fibrosis, rheumatoid arthritis, collagen vascular and asthma, as well as in inflammatory conditions including septic shock or trauma [1,2,3,4,5,6].

In addition, serum sST2 has been reported as a promising prognostic biomarker to manage cardiovascular diseases [5,7,8,9] but, as is the case with other biomarkers, sST2 is not only related to cardiovascular diseases. Recent studies have shown that sST2 can be used as a biomarker of hepatic cystic echinococcosis (CE) activity; it has been reported that it can differentially work at the cut-off value of 1246 pg mL^–1^ [10]. Moreover, the survey carried out within the Oulu Project Elucidating Risk of Atherosclerosis (OPERA) dedicated to explore the connections among cardio metabolic risk factors, different diseases, and total mortality, showed higher sST2 levels among subjects suffering from cardiovascular disease, cancer, mild cognitive decline, and diabetes, while elevated sST2 concentrations indicated a worse prognosis and could provide prognostic information on an individual mortality before any particular diagnostic [11]. In addition, it was reported that patients suffering from severity of metabolic syndrome (MetS), which comprises a group of metabolic abnormalities including central obesity, hypertension, diabetes mellitus (DM) or hyperglycaemia, high triglyceride (TG) levels, and low levels of high-density lipoprotein cholesterol (HDL-C), showed high serum sST2 levels, regardless of sex and age [12]. Additionally, the role of sST2 in pathogenesis and prognosis of different types of cancer, such as glioblastoma [13], breast cancer [14], pancreatic cancer [15], and leukaemia [16] has been described. ST2 is considered as a key molecule regulating cell proliferation [6] and exerting a pro-tumorigenic effect on diverse types of cancer, including breast, colon, liver, lung, and pancreatic cancers, among others [17]. Moreover, high ST2 expression has been associated with poor survival and it is considered as a potential target for colorectal cancer immunotherapy [18]. A study with chemotherapy-treated advanced pancreatic ductal adenocarcinoma (PDAC) patients showed that sST2-plasma levels lower than 13,064 pg mL^–1^ were related to higher overall survival (16 months) than those over the median (4 months) [19]. Increased levels of sST2 correlating with severity have also been reported in plasma samples from patients with pancreatitis [20], a risk factor for PDAC initiation.

The evaluation of sST2 levels is generally undertaken using enzyme-linked immunosorbent assays (ELISA), sometimes limited by their low sensitivity and poor precision [5]. However, the ELISA assay reported in 2009 by Dieplinger et al. [3], known as Presage^®^ ST2, has been suggested as the only one to be used clinically [5], due to its high precision, sensitivity, and in vitro stability, with a limit of detection (LOD) <2 ng mL^–1^ [21]. Only two more methods for the determination of ST2 have been reported. One consisted of the production of molecularly imprinted polymer nanoparticles (nanoMIPs) used as synthetic antibodies for ST2 and using surface plasmon resonance (SPR), providing a LOD of 8.79 ng mL^–1^ [22]. The second method was an impedimetric immunosensor using a fullerene C60-modified disposable graphite paper electrode allowing a low LOD of 0.124 fg mL^–1^. However, the impedimetric immunosensor needed more than 14 h for preparation, followed by 30 min for antigen incubation before measurement [23].

In this paper, we describe a simple, sensitive, specific, precise, and fast electrochemical immunoassay for the determination of ST2. The method was successfully applied to the analysis of sST2 in human plasma from healthy individuals and from patients diagnosed with PDAC. The target analyte in the plasma samples was specifically captured with immunoconjugates prepared on the surface of MBs, sandwiched with a biotinylated detector antibody (btn-DAb), and labelled with a Strep-HRP conjugate. SPCEs were used as amperometric transductors to detect the activity of the enzyme magnetically captured at the surface of the working electrode in the presence of H_2_O_2_ and hydroquinone (HQ).

## 2. Materials and Methods

### 2.1. Apparatus and Electrodes

A CHI812B potentiostat (CH Instruments, Inc., Austin, TX, USA) operated by the software CHI812B was used for the electrochemical measurements. Screen-printed carbon electrodes (SPCEs, DRP-110) and the mandatory cable connector (DRP-CAC) were purchased from Methrom Hispania, S.I., (Madrid, Spain). A magnetic concentrator (DynaMag™-2, 123.21D, Invitrogen Dynal AS, Carlsbad, CA, USA) was used to efficiently separate and handle MBs. Other instruments employed include: a constant temperature incubator shaker (Optic Ivymen^®^ System, Comecta S.A, Scharlab, Madrid, Spain), a magnetic stirrer (Inbea S.L.), a Vortex Bunsen AGT-9, a Basic pH-meter (Basic 20+, Crison, Barcelona, Spain), and a homemade polymethacrylate (PMMA) casing with a neodymium magnet (AIMAN GZ) embedded. ELISA flat-bottom plates and a Magellan V 7.1 (TECAN) ELISA plate reader were used to compare the results obtained with the developed electrochemical immune platform.

### 2.2. Materials and Reagents

MBs functionalized with carboxylic acid groups (HOOC-MBs, 2.7 µm Ø 10 mg mL^–1^, Dynabeads^®^ M-270 carboxylic acid, Cat. No: 14305D) were acquired from Invitrogen-Thermo Fisher. Furthermore, 2-(N-morpholino) ethanesulfonic acid (MES), sodium chloride, potassium chloride, sodium dihydrogen phosphate, di-sodium hydrogen phosphate, and Tris-hydroxymethyl-aminomethane-HCl (Tris-HCl) from Scharlab were used. N-(3-dimethyl-aminopropyl)-N’-ethylcabodiimide (EDC), N-hydroxysulfosuccinimide (Sulfo-NHS), ethanolamine, Tween^®^ 20, hydroquinone (HQ), and hydrogen peroxide (H_2_O_2_, 30% w/v) were purchased from Sigma-Aldrich. Blocker casein solution (BB) (consisting of a 1% w/v purified casein PBS solution, pH 7.4) from Thermo Scientific was used. Anti-ST2 murine monoclonal capture antibody (anti-ST2-CAb), recombinant human ST2 standard, and goat anti-human ST2 detector antibody modified with biotin (btn-DAb) were used; all of which were included in the Human ST2/IL-33R DuoSet^®^ ELISA (Cat. No: DY523B-05, R&D Systems, Inc.). Human hemoglobin (Hb), human serum albumin, (HSA) and IgG from human serum were purchased from Sigma-Aldrich. Streptavidin peroxidase conjugate (Strep-HRP) from Roche Diagnostics GmbH was used.

Next, 0.05 M of pH 6.0 phosphate buffer, 0.1 M of pH 8.0 phosphate buffer, pH 7.4 phosphate-buffered saline (PBS), 0.025 M of pH 5.0 MES buffer, and 0.1 M of pH 7.2 Tris−HCl buffer solutions were prepared with deionized water from a Millipore Milli-Q purification system (18.2 MΩ cm).

### 2.3. Preparation of the Magnetic Immunoconjugates and Electrochemical Readout

The protocol for the biofunctionalization of the MBs was carried out under continuous stirring (950 rpm) at 25 °C. A 3 µL-aliquot of the commercial HOOC-MBs solution was deposited into a 1.5 mL centrifuge tube and, after the two washing steps with 50 µL of MES buffer for 10 min, the surface carboxylic groups of the MBs were activated with 25 µL of a 50 mg mL^–1^ mixture solution of EDC/Sulfo-NHS prepared in MES buffer for 35 min. Once activated, the beads were washed twice with 50 µL MES buffer and incubated with 25 µL of a 10 µg mL^–1^ anti-ST2-CAb solution in MES buffer for 15 min. Thereafter, the blocking of the activated free remaining MBs sites was made with 25 µL of 1 M ethanolamine solution prepared in 0.1 M of phosphate buffer at a pH of 8.0, for 60 min. The modified beads were then washed twice with 50 µL of 0.1 M Tris-HCl buffer at a pH of 7.2, and once more with 50 µL of BB. Subsequently, they were incubated in a 25 µL-aliquot of human ST2 standard (or the properly diluted plasma sample to be analyzed) in BB for 15 min. Thereafter, the modified beads were washed twice with 50 µL of BB and incubation with 25 µL of a mixture solution containing 1.0 µg mL^–1^ btn-DAb and 1:1000 diluted Strep-HRP conjugate, prepared in BB, and accomplished for 30 min to form the sandwich immunocomplexes. Two latter additional washings with 50 µL BB were finally made. 

The as-modified MBs were re-suspended in 0.05 M of pH 6.0 sodium phosphate buffer (50 µL) and magnetically concentrated by drop-casting onto the WE surface of the SPCE previously placed into the PMMA casing. The embedded magnet was positioned just below the carbon working electrode of the SPCE to ensure the reproducible capture of the modified MBs onto the working electrode surface. The ensemble SPCE/magnet holding block was connected to the electrochemical station through the cable connector and immersed into an electrochemical cell containing 10mL of the same buffer solution and 1.0 mM of freshly prepared HQ. Amperometric readings in stirred solutions were recorded at−0.20 V (vs. Ag pseudo-reference electrode) upon the addition of 50 µL of a fresh 0.1 M H_2_O_2_ solution. The provided signals correspond to the difference between the steady-state and the background currents, the error bars having been estimated as the triple of the standard deviation of three replicates (confidence intervals calculated for α = 0.05).

### 2.4. Analysis of sST2 in Plasma

The developed immunosensor was used to determine the sST2 concentration in plasma samples, both from healthy individuals and PDAC-diagnosed patients. These samples were provided by the Hospital del Mar Medical Research Institute (IMIM) and approved by the corresponding ethical permission (IMIM 2020/9067/I). Signed informed consent from all the involved subjects, were obtained.

Due to the demonstrated absence of the matrix effect, quantification was performed by simple interpolation of the amperometric responses provided by the immunosensor for plasma samples diluted 25-fold or 50-fold for healthy subjects or PDAC patients, respectively, into the calibration plot constructed with ST2 standards.

In addition, the same samples were analyzed by applying the ELISA method which used the same immunoreagents according to the previously reported protocol [24] with minor modifications (1.0 µg mL^−1^ anti-ST2-CAb solution, prepared in PBS; 200 ng mL^−1^ btn-DAb solution (in Reagent Diluent solution), and Strep-HRP prepared by diluting 40 times with Reagent Diluent solution the solution provided in the commercial kit). The absorbance values obtained for plasma samples that were 25-times diluted from healthy individuals and 100-times diluted from PDAC patients were interpolated into the calibration graph constructed with ST2 standards.

## 3. Results

**Figure 1:**
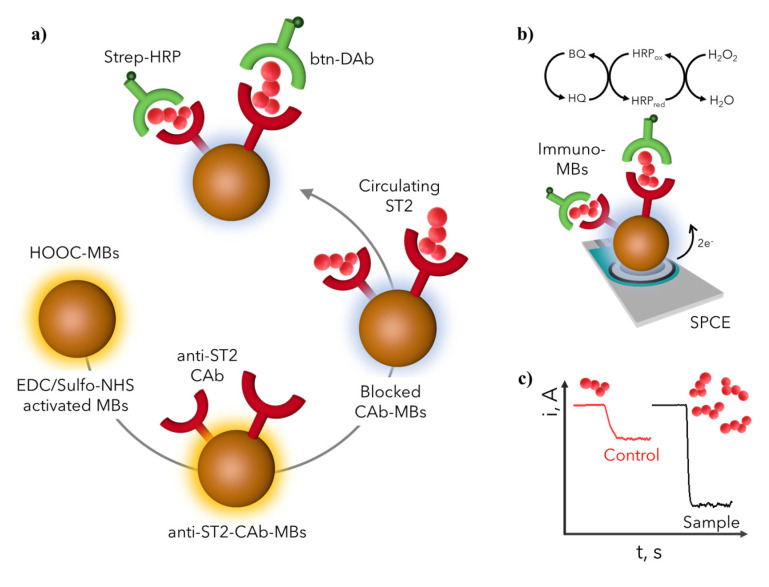
Preparation of the MB-based immunosensor for the determination of ST2. (**a**) Steps involved in the sandwich immunoassay; (**b**) Electrochemical transducer and reactions involved in the amperometric readout; (**c**) Example of amperometric traces recorded for control and high ST2 content samples.

Figure 1a schematically shows the developed platform involving magnetic immunocarriers prepared by the covalent immobilization of a specific capture antibody (anti-ST2-CAb) onto HOOC-MBs, which selectively capture ST2. This protein was subsequently recognized with a specific biotinylated detector antibody (btn-DAb) which was enzymatically labeled with a (Strep-HRP) polymer. Amperometric detection at –0.20 V (vs. Ag pseudo-reference electrode) was performed onto SPCEs where the MBs bearing the sandwich immunocomplexes were magnetically located using a magnet-holding block. The bioaffinity reactions taking place on the surface of the immunocarriers were evaluated in the presence of HQ as a redox mediator, using H_2_O_2_ as an enzymatic substrate (Figure 1b). The recorded cathodic current was proportional to the target protein level (Figure 1c).

### 3.1. Optimization of Experimental Variables

**Figure 2:**
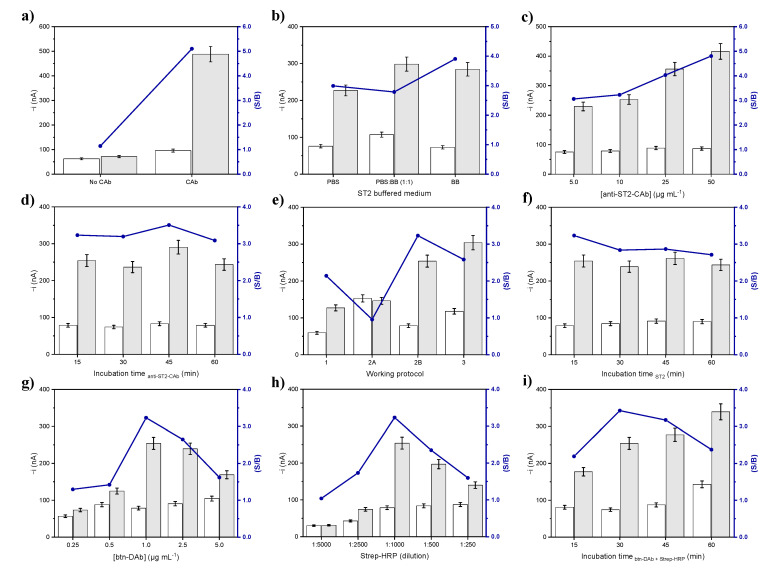
Feasibility of the immunosensor design (**a**), and effect of the working variables on the amperometric response provided by the developed immune platform (**b–i**): (**b**) different buffered medium for the preparation of ST2 standard solutions; (**c**) concentration and (**d**) incubation time of anti-ST2-CAb; (**e**) steps involved in the working protocol; (**f**) incubation time with ST2; (**g**) concentration of btn-DAb; (**h**) Strep-HRP dilution; and (**i**) incubation time of the mixture containing btn-DAb and Strep-HRP. Amperometric responses measured in the presence of 0.0 (white bars) and 1000 (**a**) or 500 (**b–i**) (grey bars) pg mL^–1^ ST2 and the resulting signal-to-blank ratios ((S/B), blue dots and lines).

The fundamentals of the proposed immune platform were verified by comparing the amperometric responses measured using both unmodified MBs and anti-ST2-CAb-MBs in the presence of 0.0 (B) and 1000 pg mL^–1^ (S) ST2 standards, as well as the corresponding S/B ratio values. To ensure that the variation in the S/B ratio values was related only to the presence and concentration of ST2, the same buffer solution (BB) was used to perform both S and B measurements. Figure 2a confirmed negligible non-specific adsorptions of ST2, Strep-HRP and btn-DAb when no specific anti-ST2-CAb was attached onto the HOOC-MBs, confirming the rationale of the proposed sandwich-based immunoassay. 

The main experimental variables involved in the preparation and performance of the immune platform were optimized. The comparison of the amperometric measurements (–0.20 V *vs.* the Ag pseudo-reference electrode) in the absence (B) and in the presence of 500 pg mL^–1^ (S) ST2, according to the S/B ratio, was taken as the selection criterion. The amount of magnetic micro-carriers and the variables involved in the amperometric detection, including the detection potential, the pH, and composition of the supporting electrolyte and the concentrations of H_2_O_2_ and HQ, were the same as those optimized in previous works [25,26]. The selected pH value of 6.0 [26] agrees with the optimum pH range (6.0–6.5) reported for HRP [27]. The study of the applied potential influence on the amperometric detection at SPCEs using the HRP/H_2_O_2_/HQ system in 0.05 M of pH 6.0 sodium phosphate buffer led to the selection of a potential value of−0.20 V as appropriate to achieve good sensitivity and precision [25,26].

The tested ranges for each variable as well as the selected values are summarized in Table 1, while the obtained results are displayed in Figure 2b–i.

Figure 2b shows the effect of the buffer solution composition on the ST2 recognition and non-specific adsorptions. BB, PBS, and a (1:1) PBS:BB mixture were tested, with a larger S/B ratio observed when using BB, which was selected as a buffer medium to carry out the assay.

The amperometric responses in the presence of ST2 increased with the concentration of the specific anti-ST2-CAb solution to be immobilized onto the surface of the MBs, while there was no significant variation in the B response (Figure 2c). Nevertheless, a concentration of 10 µg mL^–1^ anti-ST2-CAb was chosen for further work because of the sufficiently large S/B ratio achieved and to improve the assay affordability. It is clear that if higher sensitivity for ST2 determination was required, 50 µg mL^–1^ anti-ST2-CAb can be used. As it is observed in Figure 2d, an incubation time of 15 min was enough for the immobilization of the anti-ST2-CAb onto the activated MBs. 

The number of steps involved in the assay was optimized by checking different working protocols, requiring different stages of incubation in solutions of different composition, all of these started from the prepared anti-ST2-CAb-MBs: (1) a protocol involving three successive incubation steps with the ST2 standard (30 min), btn-DAb (30 min), and Strep-HRP (15 min) solutions; (2A) a protocol involving two successive steps involving incubations in a mixture solution containing ST2 standard and btn-DAb (30 min), and in a Strep-HRP solution (30 min); (2B) a protocol involving two successive steps consisting of incubation with the ST2 standard solution (30 min) followed by another incubation in a btn-DAb/Strep-HRP mixture solution (30 min); and (3) a protocol involving a single incubation in a mixture solution containing the ST2 standard, btn-DAb, and Strep-HRP (30 min). Figure 2e shows that larger S/B ratios were reached for protocols 2B and 3, probably due to an improved efficiency in biorecognition events when the reagents are in homogeneous solution. As evidenced, a better S/B ratio was reached when ST2 was first captured on the anti-ST2-CAb-MBs, and subsequently incubated in a btn-DAb and Strep-HRP mixture solution (Figure 2e, bars 2B). From this result we can deduce that when target ST2 is firstly captured by the detector antibody, the effect of steric hindrance of the ST2-btn-DAb complex is more apparent in hampering the efficient recognition by the anti-ST2-CAb modified MBs. Therefore, this two-step working protocol (protocol 2B) was selected for further work. According to this optimized protocol, the time required for the complete modification of the MBs, and therefore for the preparation of the biosensor and the determination, is 175 min. However, it should be noted that the biosensor can be prepared, or the determination performed in as little as 45 min if starting from anti-ST2-CAb-MBs, which, according to the results discussed in Section 3.2, are stable for at least 22 days.

Figure 2g,h display the effect of btn-DAb and Strep-HRP concentration on the immunosensor response, respectively. The S/B ratio increased with btn-DAb concentration up to 1.0 µg mL^–1^ and progressively decreased for larger concentrations due to the slight increase in the blank signal (B) and the decrease in the specific response in the presence of ST2 (S), which can be explained because of a hindered molecular recognition in the presence of large btn-DAb amounts. As expected, smaller S/B ratio values were obtained when low Strep-HRP tracer was loaded onto the modified MBs (Figure 2h). According to Figure 2f and Figure 2i, incubation times of 15 and 30 min, were selected for capturing ST2 and for its simultaneous recognition and labeling through the HRP-Strep-btn-DAb mixture, respectively.

### 3.2. Calibration Curves and Analytical Characteristics of the Immune Platform

**Figure 3:**
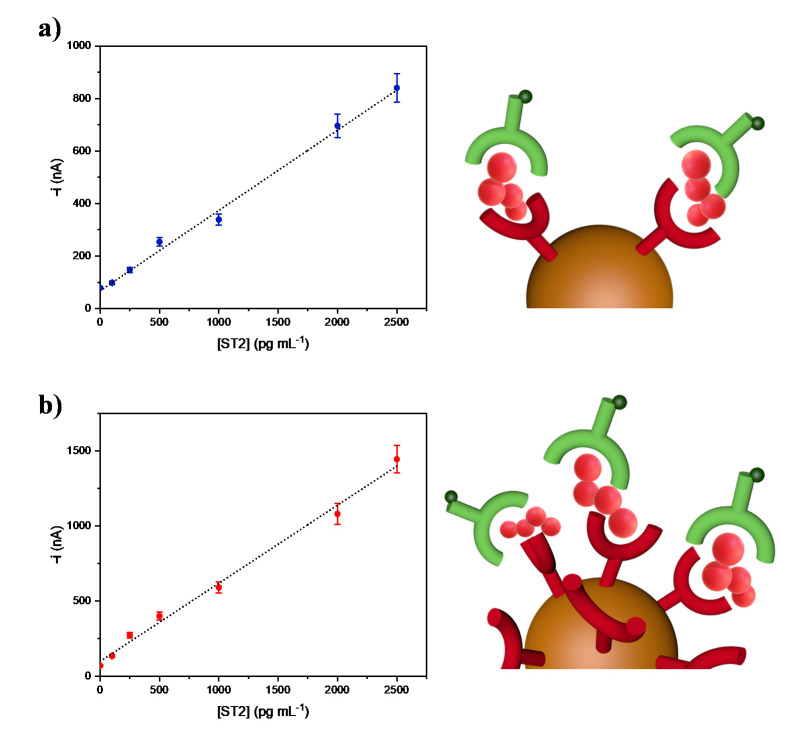
Calibration curves constructed for the amperometric determination of ST2 with immune platforms prepared from MBs modified with (**a**) 10 µg mL^–1^ or (**b**) 50 µg mL^–1^ anti-ST2-CAb solutions. Comparative fictitious pictures of the anti-ST2-CAb loading onto the magnetic immunoconjugates are also shown.

Calibration graphs for ST2 constructed with immune platforms were prepared with either a 10 or 50 µg mL^–1^ capture antibody and loaded onto the magnetic microcarriers (Figure 3a,b).

The amperometric responses increased with the ST2 concentration over the 141 to 2500 pg mL^–1^ and 76 to 2500 pg mL^–1^ ranges for the immunosensors prepared using 10 or 50 µg mL^–1^ anti-ST2-CAb solutions, respectively. According to the 3×s_b_/m criterion, where s_b_ is the standard deviation for 10 amperometric measurements in the absence of ST2, and m is the slope of the corresponding linear calibration plot, LODs of 39.6 and 26.7 pg mL^–1^, respectively, were calculated. Besides, as expected, an increase in the sensitivity (0.52 nA mL pg^–1^ *vs.* 0.30 nA mL pg^–1^) was apparent when using the larger loading of anti-ST2-CAb on the MBs. Importantly, both LODs values were significantly lower than the reference intervals reported for sST2 in human plasma and serum from males (11–45 ng mL^–1^ and 8.6–49.3 ng mL^-1^) and females (9–35 ng mL^–1^ and 7.32–33.5 ng mL^–1^) [28,29].

Compared with the only two (bio)sensors reported to date for ST2 determination, an extremely low LOD was claimed for the impedimetric immunosensor (0.124 fg mL^–1^). However, the method, involving disposable graphite paper electrodes coated with fullerene C60, took more than 14 h, and was applied to the analysis of spiked serum samples from healthy individuals [23]. The proof of concept SPR sensor using affinity MIP nanoparticles achieved a LOD value of 8.79 ng mL^–1^ and was just applied to the analysis of spiked fetal bovine serum [22].

**Figure 4:**
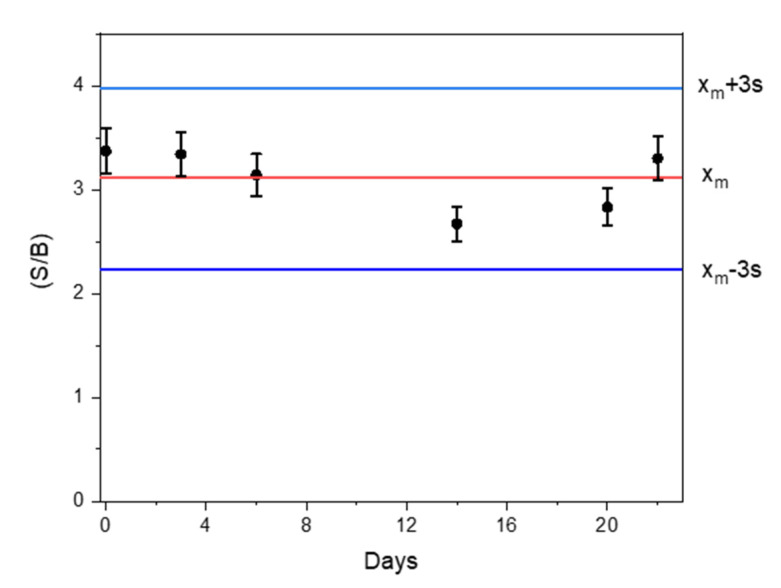
Stability of anti-ST2-CAb-MBs immunoconjugates stored in filtered PBS at 4 °C after their preparation. S/B ratio values were obtained for 0.0 and 1000 pg mL^–1^ ST2 standards with immune platforms prepared from the stored immunoconjugates each control day. Control limits (blue lines) were set as ±3 s of the mean value obtained the first day of the study (n = 3).

The developed immune platform can be prepared in 3 h without tedious synthesis procedures, and has an acceptable reproducibility (RSD value of 6.4% for the measurements of 500 pg mL^–1^ ST2 carried out with five biosensors prepared in the same manner and tested the same day), and great stability (no significant differences in terms of S/B ratio values were observed for amperometric measurements of 0.0 and 1000 pg mL^–1^ ST2 with immune platforms prepared from anti-ST2-CAb-MBs immunoconjugates which were stored in filtered PBS at 4 °C for at least 22 days, as seen in Figure 4).

ASPECT-PLUS ST2 test, a commercially available cassette (Critical Diagnostics), allows the performance of a point-of-care test (POCT) for the determination of sST2 in human plasma within a concentration range from 12.5 to 250 ng mL^–1^ [30]. This fast (less than 35 min), quantitative lateral flow immunoassay makes use of murine mouse monoclonal antibodies against human ST2, and goat polyclonal antibodies against murine IgG performing fluorescence detection. Moreover, Dieplinger et al. performed a clinical comparison of the ASPECT-PLUS ST2 test, MBL and PRESAGE ST2 ELISAs. These authors concluded that, despite ASPECT-PLUS meeting the analytical requirements for POCT and providing comparable results to those obtained with the PRESAGE ST2 ELISA kit, there were large variation coefficients, close to 17%, and a considerable high LOD (about 12 ng mL^–1^), and thus that ASPECT-PLUS is probably not suitable for risk stratification of healthy and/or population-based cohorts with endogenous sST2 levels below this concentration [31]. PRESAGE ST2 and MBL ST2 sandwich-based ELISA kits provided LODs of 1.3 ng mL^–1^ and 32 pg mL^–1^, respectively, which are comparable to that achieved with the developed amperometric immune platform. However, ELISAs have important comparative limitations in terms of multiplexing ability and portability.

### 3.3. Selectivity of the ST2 Immune Platform

**Figure 5:**
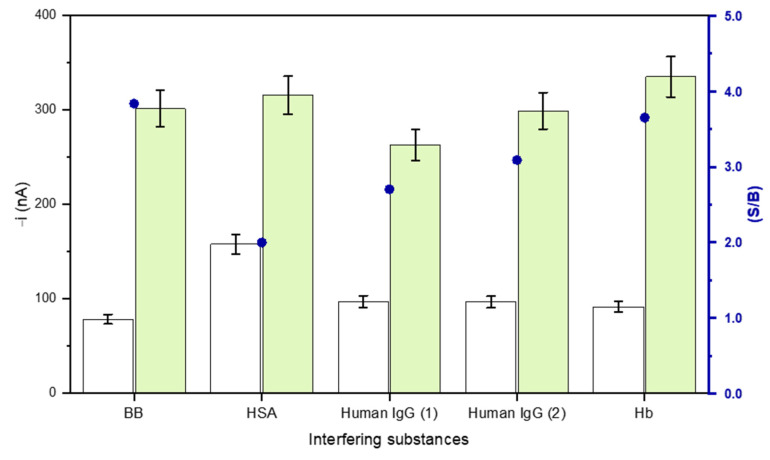
Amperometric measurements carried out with the developed immunosensor in the presence of different serum proteins: 0.0 (white bars) and 1000 pg mL^–1^ (green bars) ST2 standards (and the corresponding S/B ratio, blue dots) prepared in the absence or in the presence of 5.0 mg mL^–1^ HSA, 1.0 and 0.1 mg mL^–1^ human IgG (human IgG (1) and (2), respectively) and 5.0 mg mL^–1^ Hb.

The responses of the developed immunosensor were checked in the presence of some potential interfering proteins usually found in serum. Amperometric responses were measured for 0.0 (B) and 1000 pg mL^–1^ ST2 (S) in the absence and in the presence of 5 mg mL^–1^ HSA, 1.0 and 0.1 mg mL^–1^ human IgG, and 5 mg mL^–1^ Hb. Figure 5 shows that 1.0 mg mL^–1^ human IgG and HSA significantly affected the immunosensor response. As it is well known, the presence of human anti-mouse antibodies (HAMAs) leads to significant errors in sandwich assay configurations using murine monoclonal antibodies, due to the possible cross-link between them and as they both capture and label detector antibodies in the absence of a target analyte [32]. The interference in the presence of HSA was also observed in an electrochemical aptasensor for the determination of MUC1 [33], as well as between HSA and monoclonal immunoglobulins [34]. It appears that the degree of interference of HSA in immunoassays may be conditioned by its degree of purification, which depends on the presence of IgGs with a wide range of specificities that may perturb the assay.

Moreover, according to specifications of the DY523B-05 DuoSet^®^ ELISA, IL-1α, IL-1β, IL-1rα, IL-1RAcP/Fc chimera, IL-1 RI, IL-1 RII and recombinant mouse ST2/Fc chimera do not exhibit cross-reactivity with the antibodies involved in the immunosensor at a concentration level of 50 ng mL^–1^, and the recombinant human IL-33 only interferes at concentrations above 97.7 pg mL^–1^.

It is important to note that, as it is shown in the next section, the interferences observed for high human IgG and HSA did not hinder the reliable determination of sST2 in plasma.

### 3.4. sST2 determination in Plasma

**Figure 6:**
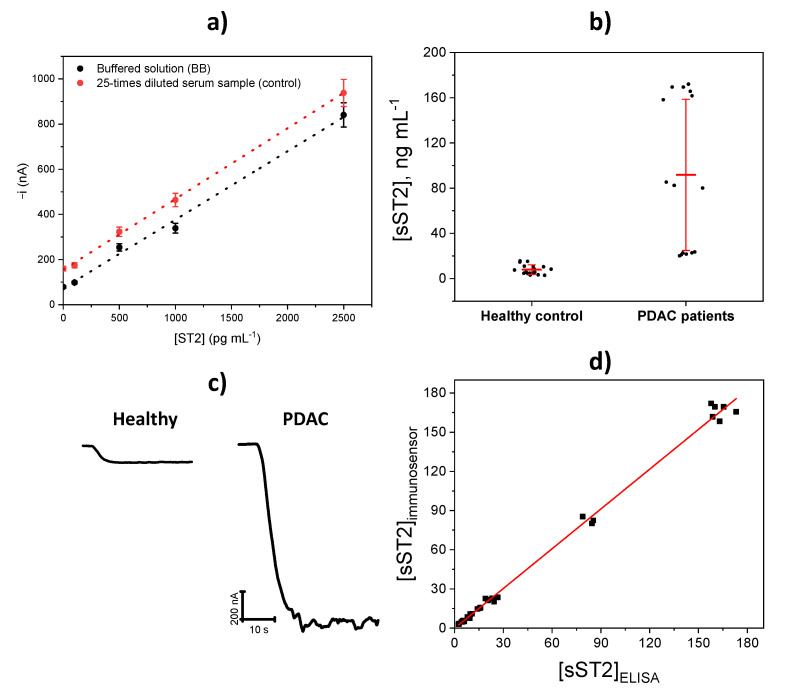
(**a**) Comparison of the calibration plots constructed with ST2 buffered standard solutions and by adding ST2 standards to a 25-times diluted plasma sample from a healthy individual. (**b**) Concentration of sST2 (in ng mL^–1^) obtained with the developed immune platform in plasma samples grouped into pools. (**c**) Examples of the real amperometric traces recorded with the immune platform for diluted plasma samples (25-times for healthy individuals and 50-times for PDAC patients) for samples 6 (healthy individual) and 9 (PDAC patient) of Table 2. (**d**) Correlation plot for the sST2 concentration values provided by the developed immune platform and the ELISA method (individual replicates performed for the determinations in the 11 plasma samples are included in the plot).

The determination of sST2 was carried out in plasma from healthy individuals and patients diagnosed with PDAC. The possible matrix effect was assessed by comparing the slope values of the calibration plots prepared with ST2 buffered standard solutions (0.30 ± 0.01 nA mL pg^–1^) using 10 µg mL^–1^ anti-ST2-CAb, and by adding ST2 standards to a 25-times diluted plasma sample from a healthy individual (0.31 ± 0.06 nA mL pg^–1^) (Figure 6a). The values calculated using the Student’s t-test (t_exp_ = 0.335 < t_tab_ = 2.571) showed no significant differences between the slope values for both calibration curves, which means that there were no matrix effects in the diluted sample.

In this regard, the analysis was done by interpolating the amperometric readings for 25 (healthy individuals) or 50 (PDAC patients) times diluted plasma samples into the calibration constructed with standards. Different dilutions were applied to the samples due to the significantly higher concentration of sST2 in those from oncology patients with the aim of obtaining amperometric signals around the midpoint of the linear calibration plot constructed with ST2 standards to minimize the error in the concentration quantification by interpolation. The obtained results are summarized in Table 2, which also includes the results provided by the ELISA methodology for the same samples according to the protocol described in Section 2.4.

Figure 6b shows the results obtained with the immune platform by patient pool. Figure 6c displays representative amperograms from plasma samples of healthy individuals and PDAC patients. In addition, the correlation plot of the results provided by both methodologies are shown in Figure 6d.

As expected, larger average sST2 concentrations were found for PDAC patients. Remarkably, two patients with larger sST2 concentrations (samples 7 and 10 in Table 2) suffered from high-grade metastatic tumors (stage 4) and showed worse prognosis (4.06 and 0.63 months, respectively). These results agree well with data reported by Kieler et al. [19], indicating a significant negative association of sST2 levels in plasma with the median overall survival rates (mOS). 

In addition, the statistical comparison shown in Table 2 (t_exp_ < t_tab_ = 2.776) and the correlation parameters (slope (1.02 ± 0.02); intercept (0 ± 1) ng mL^–1^; R^2^ =0.996) confirmed the absence of significant differences between the results provided by the developed immune platform and the ELISA method, which is incompatible with decentralized and multiplexed determinations.

## 4. Conclusions

This work reports a novel immune platform, involving the use of MBs and disposable carbon electrodes, for the simple and rapid (45 min) determination of ST2. Sandwich immunocomplexes labeled with HRP were prepared on the MBs surface and amperometric transduction was performed, upon MBs magnetic capture on the surface of SPCEs. The immune platform exhibits a high sensitivity (LOD value in the low pg mL^–1^ level) and a selectivity compatible with the clinical application. In fact, the immune platform was successfully applied to the analysis of plasma samples from patients diagnosed with PDAC upon a simple dilution and without matrix effect. The obtained results clearly confirmed the usefulness of the developed immune platform and the good agreement with those provided by the ELISA method, thus allowing for the minimally invasive diagnosis of PDAC.

Importantly, considering the reported data on the sST2 expression in several cancers, it is tempting to speculate that the immune platform can also be useful for the diagnosis of other tumors where this protein is overexpressed [17]. Large-scale clinical trials are needed to further evaluate the performance of this method before it can be used for definitive clinical diagnosis and/or prognosis. Nevertheless, the exhibited attributes in terms of versatility, affordable cost, and the ability to perform multiplexed determinations in an endpoint manner at POCT settings, allow envisioning the immune platform translation to the clinic to assist in the identification and/or follow-up of patients with high-prevalence and mortal cancer diseases.

## Figures and Tables

**Table 1:** Assessed experimental variables optimized for the ST2 immunosensor development.

Variable	Tested Range	Selected Value
ST2 standard buffered medium	PBS; PBS:BB; BB	BB
[anti-ST2-CAb] (µg mL^–1^)	0.0–50	10
Incubation time _anti-ST2 CAb_ (min)	15–60	15
Number of steps for the assay	1–3	2
Incubation time _ST2_ (min)	15–60	15
[btn-DAb] (µg mL^–1^)	0.25–5.0	1.0
Strep-HRP dilution	1:250–1:5000	1:1000
Incubation time _btn-DAb + Strep-HRP_ (min)	15–60	30

**Table 2:** Concentration of sST2 (in ng mL^–1^) obtained from the measurements carried out with the immune platform and the ELISA method in plasma samples.

Subjects	Sample	Immune Platform		ELISA		t_exp_	t_tab_
		[sST2] ^1^	RSD_n = 3_, %	[sST2] ^1^	RSD_n = 3_, %		
Healthy individuals	1	(5.5 ± 0.8)	6.1	(5 ± 1)	9.3	0.009	2.776
	2	(4.7 ± 0.2)	1.3	(4.4 ± 0.7)	6.8	1.519	
	3	(15 ± 1)	3.6	(15 ± 2)	6.3	0.016	
	4	(3.1 ± 0.6)	7.7	(2.6 ± 0.5)	7.9	2.518	
	5	(10.8 ± 0.7)	2.5	(10 ± 2)	6.6	1.752	
	6	(8 ± 1)	6.0	(8 ± 4)	9.8	0.680	
PDAC patients	7	(168 ± 6)	1.3	(166 ± 28)	3.9	0.468	
	8	(22 ± 2)	3.6	(20 ± 4)	4.5	2.515	
	9	(164 ± 18)	4.4	(160 ± 12)	1.7	0.933	
	10	(83 ± 6)	3.2	(83 ± 15)	4.3	0.098	
	11	(22 ± 4)	8.1	(25 ± 7)	6.9	1.767	

^1^ (mean value ± t × s/√n, n = 3).

## Data Availability

The data that support the findings of this study are available from the corresponding authors upon reasonable request.
